# STIM1 activation is regulated by a 14 amino acid sequence adjacent to the CRAC activation domain

**DOI:** 10.3934/biophy.2016.1.99

**Published:** 2016-02-28

**Authors:** Marek K. Korzeniowski, Barbara Baird, David Holowka

**Affiliations:** Department of Chemistry and Chemical Biology Cornell University, Ithaca, NY 14853

**Keywords:** CRAC channel protein1 (ORAI1), cell signaling, fluorescence resonance energy transfer (FRET), membrane function, stromal interaction molecule 1 (STIM1), endoplasmic reticulum (ER), conformational change

## Abstract

Oligomerization of the Ca^2+^ sensor, STIM1, in the endoplasmic reticulum (ER) membrane, caused by depletion of ER Ca^2+^ stores, results in STIM1 coupling to the plasma membrane Ca^2+^ channel protein, Orai1, to activate Ca^2+^ influx in a process known as store-operated Ca^2+^ entry. We use fluorimetry-based fluorescence resonance energy transfer (FRET) to monitor changes in STIM1 oligomerization in COS7 cells transfected with STIM1 constructs containing selected truncations, deletions, and point mutations, and labeled with donor and acceptor fluorescent proteins at either the luminal (N-terminal) or the cytoplasmic (C-terminal) ends. Our results with sequential truncations of STIM1 from the C-terminus support previous evidence that the CRAC activation domain (CAD/SOAR, human sequence 342–448) is an oligomer-promoting segment of STIM1, and they show that truncation just after CAD/SOAR (1–448) causes significantly elevated basal cytoplasmic Ca^2+^ and spontaneous STIM1 clustering. We find that a 14 amino acid sequence just C-terminal of CAD/SOAR (449–462) prevents spontaneous clustering and activation of STIM1 in COS7 cells. In response to store depletion, C-terminally labeled STIM1 without CAD/SOAR clusters together with CAD/SOAR-containing STIM1 constructs. However, these donor-acceptor pairs do not undergo a stimulated increase in FRET, exhibiting instead a decrease in FRET consistent with a stimulated conformational extension in full length STIM1. We find that the 14 amino acid sequence plays a regulatory role in this process. Overall, our FRET results provide evidence in live cells that Ca^2+^ store depletion stimulates a conformational extension in the cytoplasmic segment of STIM1 that accompanies its oligomerization.

## 1. Introduction

**Figure 1:**
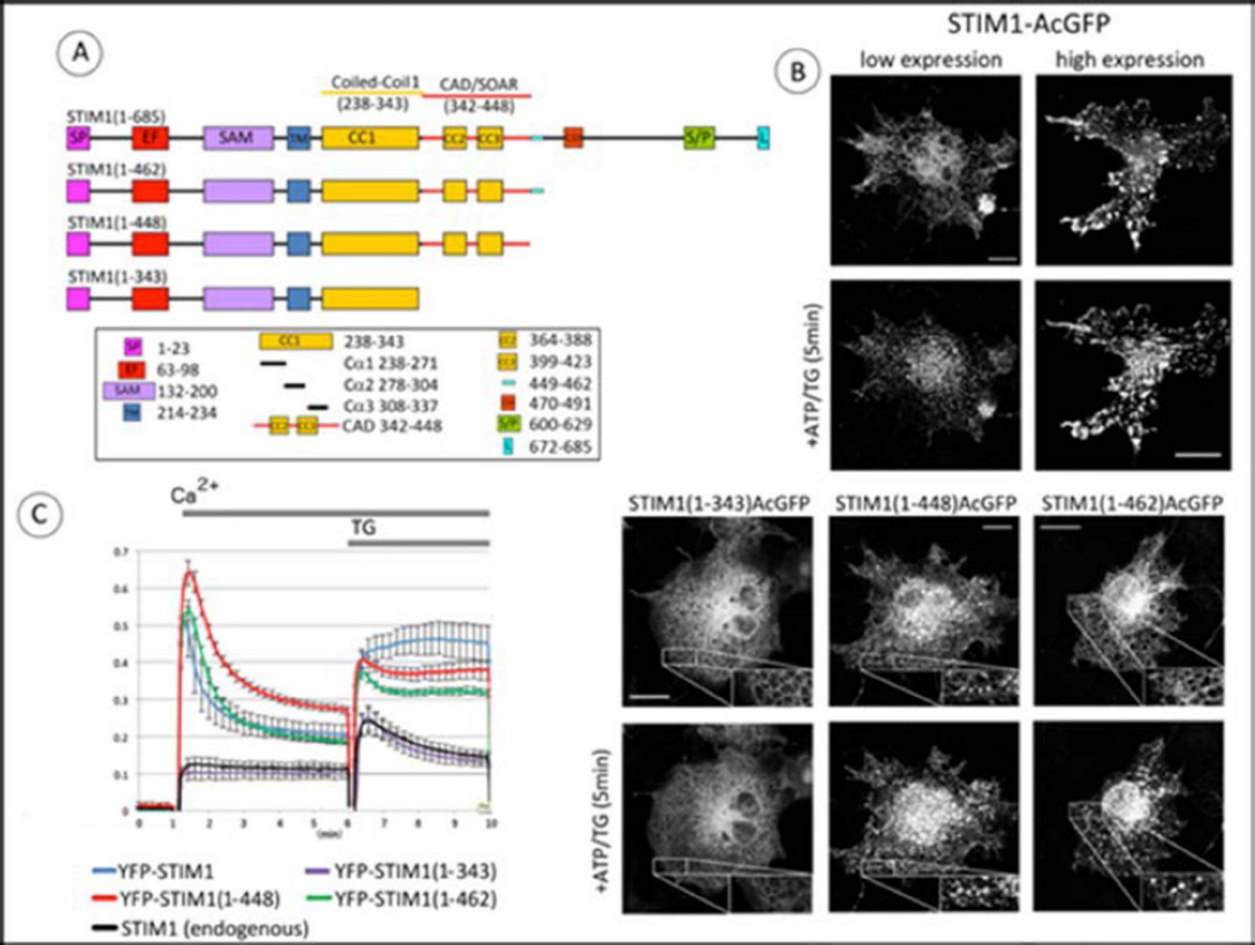
C-terminal truncation of STIM1 immediately after CAD/SOAR causes clustering of STIM1 and constitutive activation of Orai1. A) Linear map of STIM1 full length (1–685) and truncation constructs, with segments identified as: SP, signal peptide; EF, EF-hand motif; SAM, sterile alpha motif; TM, transmembrane segment; CC1/2/3, coiled-coil domains 1/2/3; CAD, CRAC Activation Domain (342–448) SOAR, STIM1 Orai1 Activation Region (344–442). CDI, Ca^2+^-dependent inactivation domain; S/P, proline/serine-rich domain; L, polybasic tail. The 14 amino acid sequence (449–462) characterized in this study is indicated by the light blue bar. B) Confocal fluorescence micrographs showing distributions of C-terminally labeled STIM1 and truncated constructs expressed in COS7 cells without Orai1. Upper four panels: low (left) and high (right) expression levels of full length STIM1-AcGFP before (top) and after (bottom) stimulation by simultaneous application of 50 µM ATP and 200 nM thapsigargin (TG) to deplete Ca^2+^ stores. Lower six panels: Truncation mutants STIM1(1–343)-AcGFP, STIM1(1–448)-AcGFP, and STIM1(1–462)-AcGFP before (top) and after (bottom) stimulation. Insets show magnification of indicated regions. Scale bars are 20 µm. C) Basal and stimulated Ca^2+^ levels in COS7 cells transfected with Orai1 and co-transfected with N-terminally YFP-labeled STIM1 constructs: full length STIM1, STIM1(1–462), STIM1(1–448), or STIM1(1–343). Transfected cells were suspended in Ca^2+^ free medium before addition of Ca^2+^ to 2 mM for 5 min; then Ca^2+^ store depletion was elicited by addition of 200 nM TG as indicated by gray bar. Experiments were performed at 37 °C. Data are the averages of 4–5 independent experiments, and error bars indicate SEM.

The molecular basis for store-operated Ca^2+^ entry (SOCE) has been extensively investigated since the discoveries of the ER Ca^2+^ sensor, STIM1 (and its homologue, STIM2; [1,2]) and the Ca^2+^-selective channel proteins, Orai1–3, which are activated by association with oligomerized STIM1 [3–5]. Depletion of Ca^2+^ from ER stores causes oligomerization of STIM1 [6], which is necessary for productive coupling to hexameric Orai [7]. Other studies provided evidence that, in addition to oligomerization, a conformational transition in the cytoplasmic region of STIM1 is necessary for functional coupling [8–11]. Several groups found that expression of a portion of this cytoplasmic region as a soluble protein spontaneously activates SOCE [8,9,12–14]. This minimal segment, commonly referred to as SOAR (human sequence 344–442) [13] or CAD (342–448; [14]) (Figure 1A), is not active when expressed as part of full length STIM1 in the absence of store depletion, suggesting that it is normally sequestered prior to STIM1 activation and oligomerization [12,14].

In 2012, a crystal structure of the CAD/SOAR region yielded the first atomic-level resolution of this minimal active segment [15]. More recently, an NMR study revealed structural details for interactions between a trypsin-resistant STIM1 fragment that includes part of this region and the C-terminal helix of Orai1 [11], supporting participation of the basic sequence in CAD/SOAR (382–387) in this association, which was previously identified in mutagenesis studies [8,16,17]. Korzeniowski et al. [8] provided evidence for a conformational change in the cytosolic segment of STIM1 necessary for Orai1 activation and Ca^2+^ influx. Specifically, human STIM1 mutation 4EA in the Cα3 helix of the CC1 domain was found to elicit STIM1 activation [8], and two subsequent fluorescence resonance energy transfer (FRET) studies on isolated cytoplasmic segments of STIM1 provided evidence that activating mutations in the CC1 domain of STIM1 (4EA and L251S) cause structural extensions, suggesting that these conformational changes are involved in this functional STIM1-Orai1 coupling [9,10].

As described here, our study utilizes steady state fluorescence spectroscopic measurements of FRET between C-terminally labeled STIM1 proteins in live cells. Our results provide direct evidence for an activating conformational extension that occurs in wt STIM1, as well as in STIM1 truncated at residue 462 (STIM1(1–462)) in the cytoplasmic segment. In contrast, STIM1 truncated just after the CAD/SOAR region (STIM1(1–448)) is not similarly regulated, exhibiting spontaneous clustering and Orai1 activation as measured by elevated cytoplasmic Ca^2+^. These differences indicate that a 14 amino acid sequence immediately C-terminal to CAD/SOAR (449–462) plays a critical role in maintaining the STIM1 conformation in an inactive state in the absence of store depletion.

## 2. Material and Methods

### 2.1. Cloning and mutagenesis

The primers used to generate DNA constructs are listed in Table I. All N-terminally tagged human STIM1 constructs were based on pEYFP-C1 (Clontech) previously described [8]. All C-terminally tagged proteins were designed based on pEAcGFP-N1 (Clontech). The STIM1-AcGFP construct was cloned into XhoI and EcoRI restriction sites in pEAcGFP-N1, based on the strategy described for STIM1-YFP and STIM1-CFP constructs by Barr et al. [18]. The C-terminal AcGFP was then replaced with mApple within AgeI and NotI restriction sites. Site-directed mutagenesis was used to introduce desired mutations and to generate deletion constructs with Phusion® High-Fidelity DNA Polymerase (New England Biolabs). All constructs were checked by sequencing of the whole reading frame. A low expressing Orai1 construct with no fluorescent tag (TK-Orai1) was cloned into NheI and NotI restriction sites in pEGFP-N1 (Clontech), then the CMV promoter was replaced with TK (thymidine kinase promoter) using AseI and NheI restriction sites in constructs described previously [19].

### 2.2. Cell culture, transient transfection, confocal microscopy and fluorescence measurements

COS-7 cells (10^5^ cells per 35 mm dish) were transiently transfected with the indicated constructs (0.5 µg of DNA per 35 mm dish) using Lipofectamine 2000 for 24 h as described previously [20]. For confocal imaging, low expression of proteins was achieved with 10 fold less DNA per volume in MatTek coverslip dishes. Confocal measurements were performed at room temperature in a modified Krebs-Ringer buffer containing 120 mM NaCl, 4.7 mM KCl, 2 mM CaCl_2_, 0.7 mM Mg(SO_4_)_2_, 10 mM glucose, 10 mM Hepes, pH 7.4, using a Zeiss LSM 710 scanning confocal microscope and a x63/1.4 oil immersion objective. Fluorimetry-based measurements of FRET were performed at 37 °C as previously described [20].

Cytoplasmic Ca^2+^ levels were measured using an SLM 8100C steady-state fluorimeter (SLM Instruments, Urbana, IL). COS-7 cells previously transfected with R-GECO1, together with untagged Orai1 and STIM1 plasmid DNA, were harvested and suspended in modified Krebs-Ringer buffer. Cells (~10^6^/ml) were stirred in an acrylic cuvette at 37°C in Ca^2+^-free Krebs-Ringer buffer supplemented with 100 µM EGTA, and the time course of R-GECO1 fluorescence (ex 560, em 580 nm long pass filter) was monitored.

### 2.3. Statistical analysis

Calculations were performed with MS Excel and p values were calculated with two-tailed and unpaired T-test distribution functions. FRET data points were normalized based on the same day control experiments with time “0” taken one minute before activation of store depletion with ATP/TG treatment. Steady-state values of stimulated FRET responses were taken 5 min after initiation of store depletion. Positive changes in FRET correspond to increases in acceptor/donor fluorescence and negative changes in FRET correspond to decreases in this ratio.

## 3. Results

### 3.1. A 14 amino acid sequence (449–462) inhibits spontaneous clustering of STIM1

Our initial experiments evaluated the spatial distributions of C-terminally truncated forms of human STIM1 transfected into COS7 cells, which express little endogenous STIM1 and no Orai1 [21]. We compared the distributions of wild type (wt) STIM1 to truncation mutants STIM1(1–462) or STIM1(1–448), all labeled at their C-terminus with monomeric AcGFP. STIM1(1–448) includes CAD/SOAR, and STIM1(1–462) contains an additional 14 residues C-terminal to the CAD/SOAR region (Figure 1A). At low expression levels, wt STIM1-AcGFP is distributed throughout the ER in the absence of stimulation, and becomes clustered in response to store depletion by 50 µM adenosine triphosphate (ATP) together with 200 nM thapsigargin (TG), even in the absence of Orai (Figure 1B, top left panels). At high expression levels, wt STIM1 is most commonly observed in sheets independently of Orai1, and store depletion increases the size of clusters, which remain similarly localized (Figure 1B, top right panels). Other experiments showed that formation of these sheets depends on the polybasic C-terminal segment of wt STIM1 (M. Korzeniowski, data not shown). Apparent plasma membrane association of over-expressed wt STIM1 has been previously observed [22–24]. Evidence for PM-ER localization of STIM1 in unstimulated cells is shown in a recent electron microscopy study [25]. At low expression levels, STIM1(1–462)-AcGFP clusters visibly only after Ca^2+^ store depletion, similarly to wt STIM1-AcGFP. However, STIM1(1–448)-AcGFP at low expression levels forms spontaneous clusters in unstimulated COS7 cells in the absence of Orai1, and larger clusters that are observed after stimulation by ATP + TG are similarly located. A similar result was recently described with a related construct [26]. In contrast, STIM1(1–343)-AcGFP, which is truncated just before the CAD/SOAR region, exhibits general ER distribution that is not changed by stimulation (Figure 1B, lower panels). These representative distributions of STIM1 and truncated mutants suggest that the 14-amino acid sequence that follows the CAD/SOAR region (449–462) serves to regulate a structural change that promotes clustering of STIM1.

Spontaneous formation of clusters by STIM1(1–448) suggests that this truncated construct might spontaneously activate Orai1 to enable Ca^2+^ entry. We tested this by coexpressing YFP-STIM1 constructs together with untagged Orai1 and the cytoplasmic Ca^2+^ reporter, R-GECO1. In these experiments, COS7 cells were harvested in modified Krebs-Ringer buffer without Ca^2+^, then 2 mM Ca^2+^ was added, followed by 200 nM thapsigargin. As summarized in Figure 1C for multiple experiments, we found that co-expression of Orai1 together with YFP-STIM1(1–448) in COS7 cells results in an initial Ca^2+^ spike that declines over 5 min to an elevated basal Ca^2+^ level compared to YFP-STIM1(1–462) or YFP-STIM1(wt) co-expressed with Orai1, which exhibit intermediate basal Ca^2+^ levels relative to YFP-STIM1(1–343) or endogenous STIM1 together with Orai1 (Figure 1C). After thapsigargin-dependent store depletion, sustained Ca^2+^ responses are observed with all three STIM1 constructs, wt STIM1, STIM1(1–448), and STIM1(1–462), co-expressed with Orai1. For these stimulated responses, the sustained Ca^2+^ level with YFP-STIM1(1–448) is intermediate between YFP-STIM1 (wt) and YFP-STIM1(1–462). As expected, YFP-STIM1(1–343) co-expressed with Orai1 exhibits a transient stimulated response that is not significantly different from that for endogenous STIM1 with Orai1 (Figure 1C). Together, these results provide evidence that STIM1(1–448) and STIM1(1–462), co-expressed with Orai1. For these stimulated responses, the sustained Ca2+ level with YFP-STIM1(1–448) is intermediate between YFP-STIM1 (wt) and YFP-STIM1(1–462). As expected, YFP-STIM1(1–343) co-expressed with Orai1 exhibits a transient stimulated response that is not significantly different from that for endogenous STIM1 with Orai1 (Figure 1C). Together, these results provide evidence that STIM1(1–448) spontaneously activates SOCE at an enhanced level relative to similar overexpression of wt STIM1 or STIM1(1–462) which include the 14 amino acid sequence (449–462).

### 3.2. Increasing FRET between C-terminal labels in symmetric constructs measures stimulated STIM1-STIM1 oligomerization

**Figure 2:**
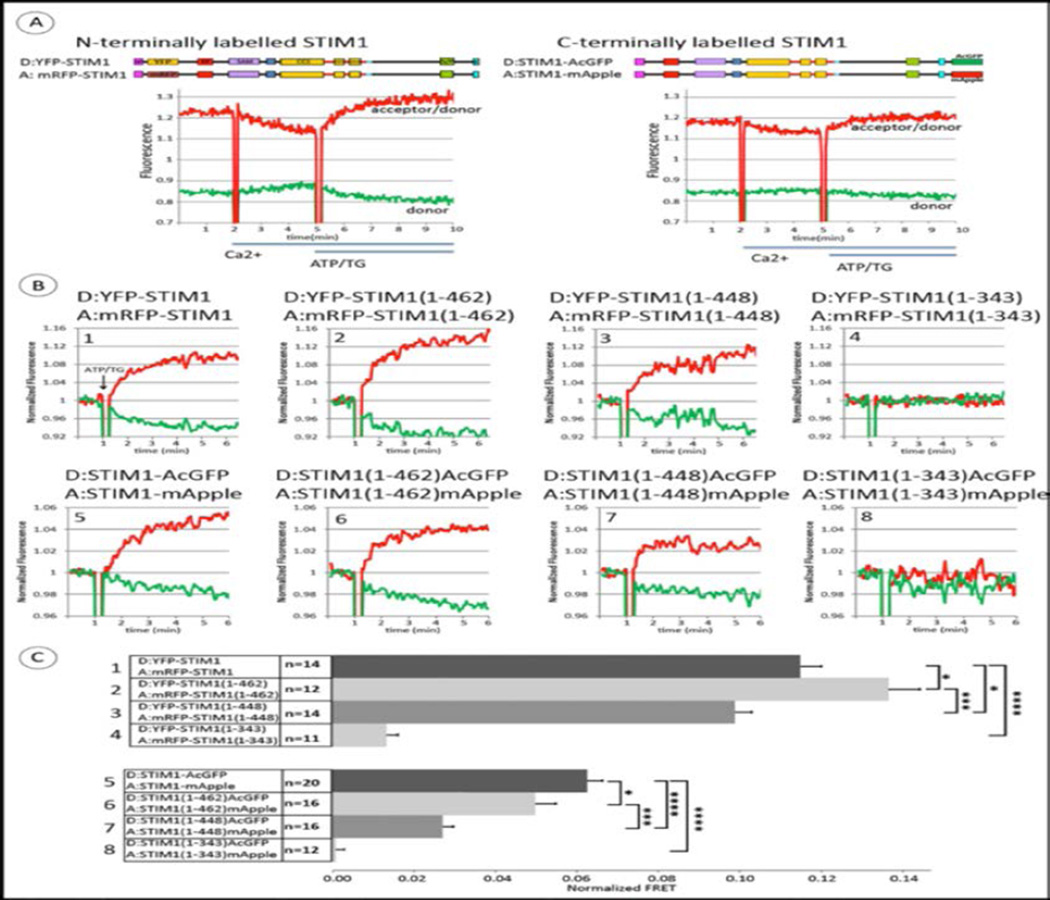
Stimulated changes in FRET between N-terminally labeled pairs (donor YFP, acceptor mRFP) or C-terminally labeled pairs (donor AcGFP, acceptor mApple) of homologous STIM1 constructs monitor oligomerization. A) Top: Linear maps of full-length STIM1 constructs, tagged on either end with donors and acceptor probes. Bottom: Time course for FRET changes in the absence of Orai1. Transfected COS7 cells were suspended initially in Ca^2+^-free medium in a fluorimeter cuvette at 37 °C, and Ca^2+^ was added to 2 mM as indicated; after 3 min, ATP+TG (50 mM/200 nM) was added to deplete Ca^2+^ stores. Representative raw data of two channel fluorimeter recordings to monitor FRET: Green trace shows donor fluorescence change, and red trace shows the ratio, acceptor fluorescence: donor fluorescence. B) Representative experiments for ATP/TG-stimulated changes in FRET between N-terminally (upper row, #1–4) and C-terminally (lower row, #5–8) tagged STIM1 constructs: Full length STIM1 (#1, 5) and STIM1 truncations: STIM1(1–462) (#2, 6), STIM1(1–448) (#3, 7) and STIM1(1–343) (#4–8). Experiments were carried out as in (A), and shown here are recordings starting at the last 2 min prior to the addition of ATP/TG (arrows). Data traces were normalized to the value of 1.0 as averaged over these last 2 min of the incubation with 2 mM Ca^2+^. Green (donor) and red (acceptor/donor) traces are the same as for (A). C) Summarized results for multiple experiments (n = 11–20) showing ATP/TG-stimulated changes in FRET between N-terminally (#1–4) and C–terminally (#5–8) tagged STIM1 constructs, with numbers corresponding to representative experiments shown in (B). Error bars are SEM with p-values calculated for compared averages as indicated: *p < 0.05, **p < 0.01, ***p < 0.001, ****p < 0.0001 (Two-tailed T-Test, unpaired).

Stimulation-dependent oligomerization of N-terminally labeled STIM1 was previously demonstrated on the nanometer scale with single cell imaging measurements of increases in FRET [6,27]. We compared FRET between N-terminally-labeled YFP-STIM1 and mRFP-STIM1 to FRET between C-terminally labeled STIM1-AcGFP and STIM1-mApple using steady-state fluorescence spectroscopy as we previously described [20]. Figure 2A (left panel) shows data for COS7 cells transfected with YFP-STIM1 and mRFP-STIM1 and pre-incubated for ~ 20 min in a Ca^2+^-free buffer. Addition of millimolar Ca^2+^ results in an increase in donor fluorescence (green) and a temporally corresponding decrease in the ratio of sensitized acceptor fluorescence to donor fluorescence (red). These changes are consistent with a decrease in FRET due to increasing separation of STIM1 proteins that occurs with reversal of oligomerization as Ca^2+^ refills the initially depleted ER stores. Subsequent stimulation by ATP + TG to fully deplete Ca^2+^ stores causes a time-dependent increase in FRET corresponding to oligomerization as previously described [6,28]. For the C-terminally labeled constructs, similar but smaller time-dependent changes in FRET occur upon initial addition of Ca^2+^ to the buffer and subsequent stimulation (Figure 2A, right panel). Although the absolute magnitude of FRET depends on the particular donor/acceptor pair, increasing FRET corresponds to decreasing distance between the donor and acceptor labels, which are symmetrically placed at the termini of these full-length constructs. These results establish our capacity to detect lateral changes in distance corresponding to STIM1 oligomerization with C-terminally-labeled STIM1 constructs and steady-state fluorimetry measurements of FRET.

We next compared the stimulated changes in FRET for both N-terminally and C-terminally labeled STIM1 proteins with the truncations described above (Figure 1A) to the FRET responses of the full length constructs. Figure 2B shows a representative set of these FRET measurements, all carried out on the same day and normalized as described in Materials and Methods to allow visual comparison of the changes in magnitude of FRET after stimulation. Raw data for a similar series of experiments showing the ratio of acceptor to donor fluorescence is shown in supplemental Figure S1. Figure 2C summarizes these results for multiple experiments, using the difference in the acceptor/donor fluorescence ratio before and after stimulation with ATP + TG as a measure of the stimulated FRET change. The stimulated increase in FRET between YFP-STIM1(1–462) and mRFP-STIM1(1–462) is somewhat larger than for the other N-terminally labeled symmetric constructs, but all exhibit some stimulated FRET, consistent with some level of stimulated oligomerization. The construct truncated at the end of CC1, just prior to CAD/SOAR (STIM1(1–343)) exhibits relatively little stimulated FRET. For the C-terminally labeled constructs, the stimulated FRET increase is greatest for wt STIM1, somewhat less for STIM1(1–462), considerably less for STIM1(1–448), with no significant stimulated FRET for STIM1(1–343) (Figure 2C). We note that although STIM1(1–448) spontaneously forms clusters as observed microscopically (Figure 1B), additional stimulated oligomerization is detected with FRET at the nanoscale, with increases for both N-terminal and C-terminal labels. However, there remains a significant difference between STIM1(1–462) and STIM1(1–448) in terms of the additional oligomerization that occurs after stimulation, consistent with the 14 amino acid sequence (449–462) playing a regulatory role in oligomerization that leads to coupling to Orai1 for Ca^2+^ uptake (Figure 1C). Little or no stimulated oligomerization or Ca^2+^ entry with STIM1(1–343) can be explained by the absence of CAD/SOAR in these constructs.

These results establish our capacity to measure stimulated oligomerization of C-terminally labeled STIM1 and to detect differences in this oligomerization that depend on the length of the cytoplasmic segment. This capacity is important for the subsequent experiments which show that these C-terminally labeled STIM1 constructs can also sense the conformational extension of the cytoplasmic segment occurs upon store depletion.

### 3.3. Decreasing FRET between C-terminal labels in asymmetric constructs measures stimulated extension in STIM1 conformation

**Figure 3:**
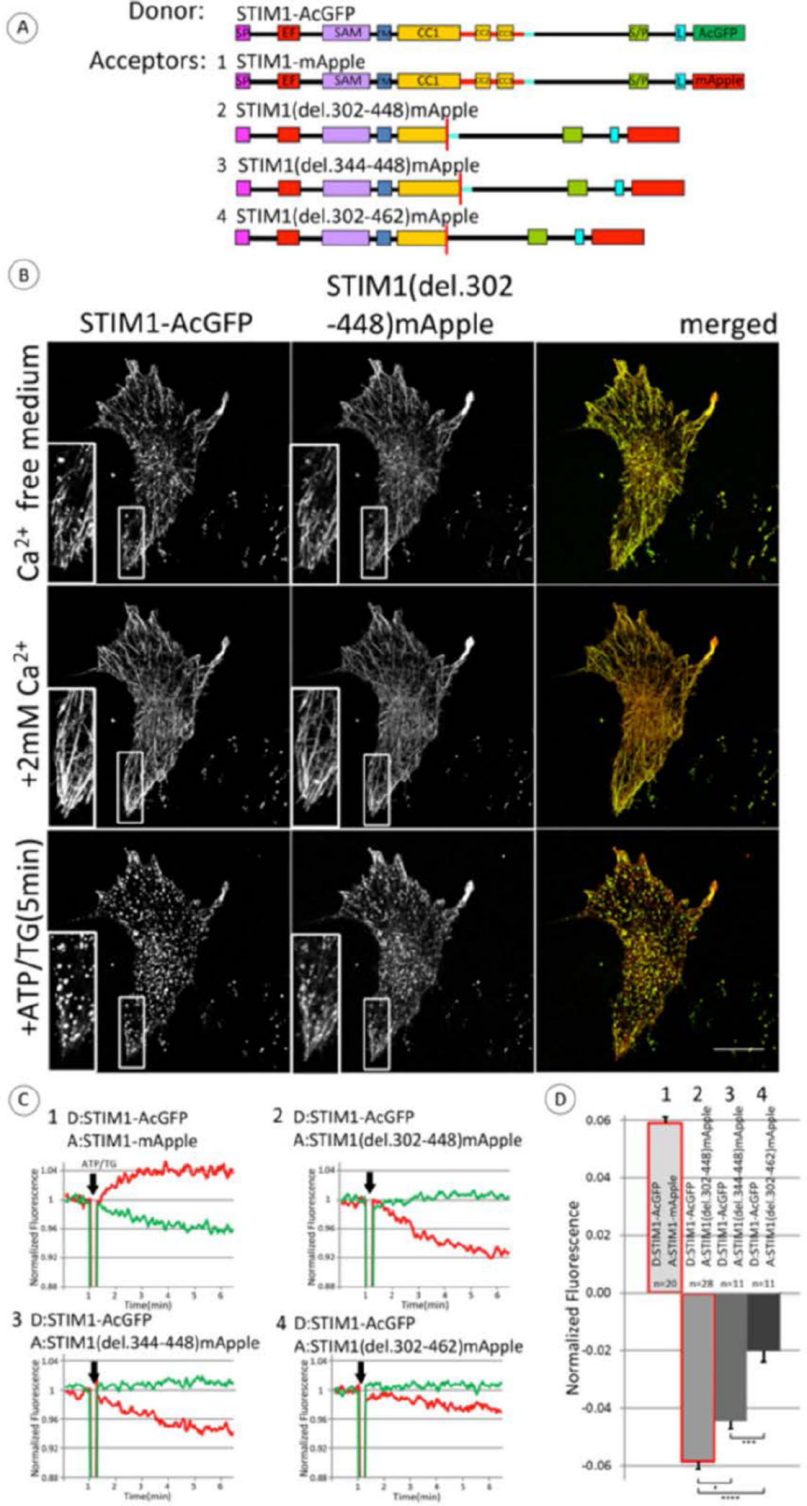
Store depletion stimulates decreased FRET between co-clustered wt STIM1-AcGFP and STIM1-mRFP mutants lacking CAD/SOAR, providing evidence for a conformational extension in STIM1. A) Linear maps of constructs used in these experiments: wt STIM1 labeled with FRET donor AcGFP on C-terminus and series of STIM deletion constructs labeled with acceptor mApple on C-terminus. Segments are labeled and color-coded as for Figure 1A; 14 amino acid sequence (449–462), normally just after CC3, is indicated with light blue bar. B) Fluorescence micrographs of COS7 cells co-transfected at low expression levels with wt STIM1-AcGFP and STIM1(del302–448)mApple and incubated for 10 min in Ca^2+^ free medium, followed by addition of Ca^2+^ to 2 mM for 10 min, then stimulation with ATP/TG (50 µM/200 nM), as indicated. Scale bar is 20 µm. Insets show clusters of STIM1(del.302–448)mApple with STIM1-AcGFP under conditions of store depletion. C) Representative experiments for ATP/TG stimulated changes in FRET between C-terminally labeled full length wt STIM1 (donor, #1–4) and STIM1 deletion constructs (acceptor) depicted in (A): #1) wt STIM1, 2) STIM1 (del302–448), 3) STIM1 (del344–448), and 4) Stim1(del302–462). Experiments were carried out and data traces are shown as in Figure 2B, with donor trace in green and (acceptor/donor) trace in red. D) Summarized results for ATP/TG-stimulated changes in FRET between C-terminally labeled full length STIM1 (donor) and STIM1 deletion constructs (acceptor), with numbers corresponding to representative experiments shown in B. Error bars are shown for SEM with p-values calculated for compared averages as indicated: * p < 0.05, **p < 0.01, ***p < 0.001, ****p < 0.0001.

We next investigated the roles of CAD/SOAR (342–448; Figure 1A) and CC1α3 (308–337; Figure 1A) in stimulated structural changes of STIM1 by deleting residues 302–448 that encompass these regions (Figure 3A). Confocal imaging shows that STIM1 (del302–448)-mApple co-clusters with wt STIM1-AcGFP in Ca^2+^-free medium; the clusters disassemble after addition of Ca^2+^ and form again upon stimulation by ATP + TG (Figure 3B). These observations indicate that STIM1 without CAD/SOAR retains the capacity to associate with wt STIM1 in response to Ca^2+^ store depletion. Interestingly, however, stimulation by ATP + TG causes a time-dependent decrease in FRET between wt STIM1-AcGFP and STIM1 (del302–448)-mApple, indicating an increase in the average nanoscale distance between the asymmetrically placed donor and acceptor labels that occurs upon store depletion. This FRET decrease is shown in Figure 3C (upper right panel), in striking contrast to the stimulated increase in FRET between wt STIM1-AcGFP and wt STIM1-mApple measured in the same experiment (Figure 3C, upper left panel). These observed differences in stimulated FRET for the asymmetrically placed donor and acceptor labels compared to those placed symmetrically (wt constructs) suggest that changes in distances occur both laterally (oligomerization state) and vertically (conformation state).

In the same experiment we also evaluated the stimulated change in FRET between wt STIM1-AcGFP and STIM1 (del344–448)-mApple, in which CAD/SOAR is deleted but CC1α3 is retained. This pair also exhibits a stimulated decrease in FRET (Figure 3C, lower left panel) that is similar to that in which both CC1α3 and CAD/SOAR are deleted (Figure 3C, upper right panel). These results are consistent with a structural extension occurring in the full length STIM1 upon stimulation that does not occur in the absence of CAD/SOAR. Interestingly, deletion of the additional 14 residues (449–462) in the acceptor construct, STIM1(del302–462)-mApple, results in stimulated FRET that also decreases, but significantly less so than when this sequence is present (Figure 3C, lower right panel). Thus, this 14 amino acid sequence immediately C-terminal to CAD/SOAR appears to strongly influence the stimulated decrease in FRET due to store depletion (i.e., the stimulated increase in distance between asymmetrically placed donor and acceptor labels) that is observed with these pairs of constructs.

These results are summarized for multiple experiments in Figure 3D. Taken together, they are consistent with the view that stimulation causes a conformational extension in the C-terminal segment of wt STIM1-AcGFP, such that the distance between the asymmetrically labeled C-termini increases, and correspondingly FRET decreases. The smaller FRET decrease observed for the acceptor construct lacking 14 amino acid sequence (449–462) in addition to CAD/SOAR + CC1α3 (302–448) suggests that this 14 amino acid sequence plays a regulatory role in the stimulated conformational extension of STIM1. In this view, the shortened STIM1(del 302–448) and STIM(del344–448) constructs undergo a more limited conformation change (and possibly oligomerization) than wt STIM1 in response to store depletion, whereas STIM1(del302–462) has a more extended conformation prior to store depletion, consistent with a role for the 14 amino acid sequence (449–462) in regulating this conformational extension. Notably, all three of these deletion constructs, as well as wt STIM1, exhibit homodimerization detected by chemical crosslinking with dithiobis(succinymidyl propionate) when separately expressed (Supplemental Figure S2), indicating that they are all capable of dimerization in the unstimulated state.

### 3.4. The 14 amino acid sequence (449–462) regulates the stimulated conformational change in STIM1

**Figure 4:**
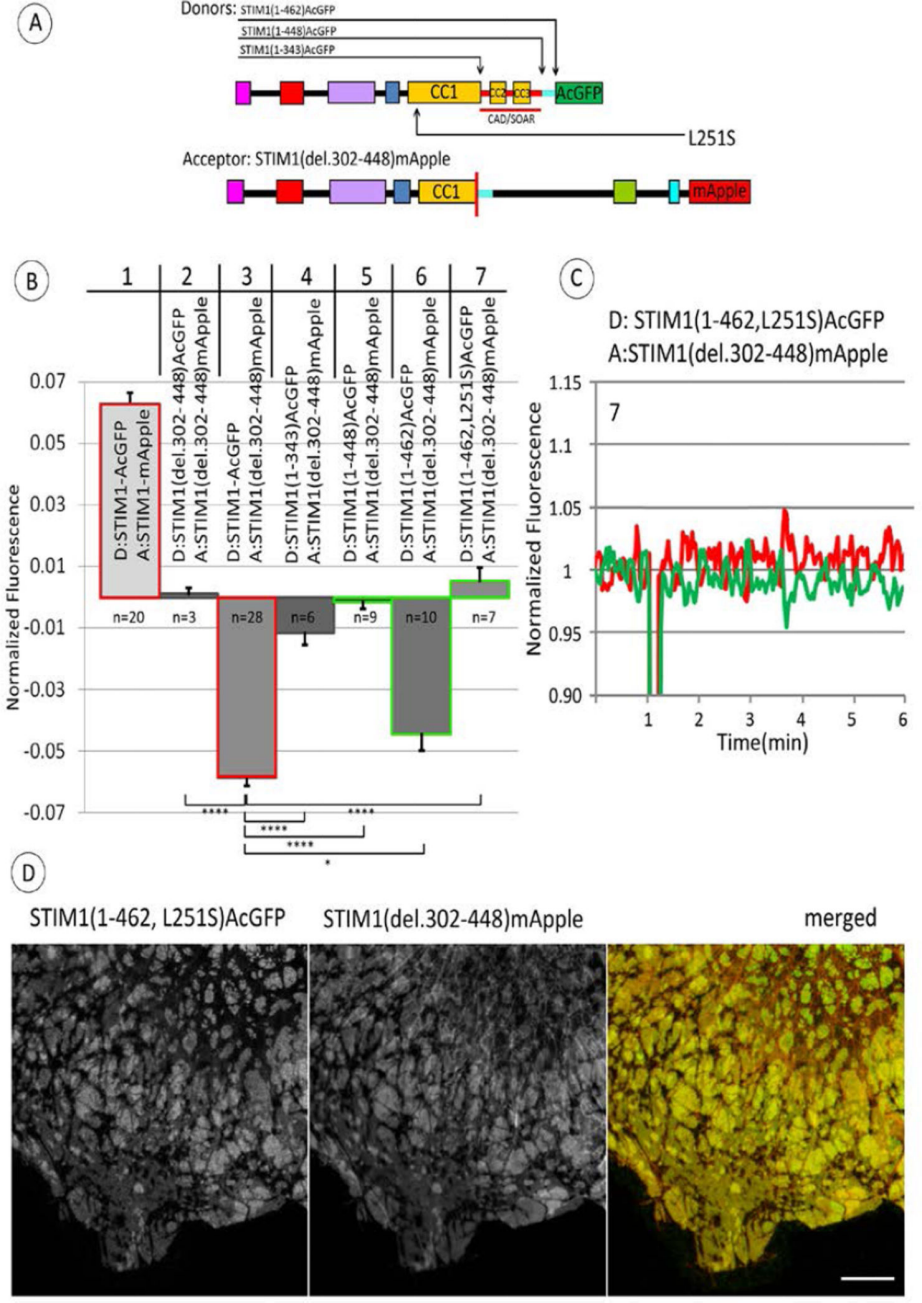
Stimulated changes in FRET between C-terminally labeled STIM1 truncation constructs (donor) and STIM1(del302–448) (acceptor) further implicate the 14 amino acid sequence (449–462) in regulating stimulated STIM1 conformational extension. A) Linear maps of constructs used in these experiments: STIM1(del302–448) labeled with FRET acceptor mApple on C-terminus, together with one of several STIM truncation constructs labeled with donor AcGFP on C-terminus. Segments are labeled and color-coded as for Figure 1A; 14 amino acid sequence (449–462), just after CC3, is indicated with light blue bar. Location of point mutation L215S is also indicated. Summarized results for ATP/TG-stimulated changes in FRET between C-terminally labeled STIM1 truncation constructs as specified (donor) and STIM1(del302–448) (acceptor). Columns #1 and 3 (outlined in red) are reproduced from Figure 3 (columns #1 and 2, respectively). Comparative FRET measurements indicating the regulatory effect of the 14 amino acid sequence (449–462) are outlined in green (columns #5, 6, 7) including that with donor STIM1(1–462, L251S) containing activating point mutation L251S (column #7). Value of n specifies number of experiments from which averages, SEM (error bars) and p values for indicated comparisons are calculated: *p < 0.05, **p < 0.01, ***p < 0.001, ****p < 0.0001. C) Representative measurement showing level of ATP/TG-stimulated change in FRET between C-terminally labeled STIM(1–462, L252S) (donor) and STIM1(del302–448) (acceptor). Experiment was carried out as described in Figure 2A with recordings shown as described in Figure 3C. This experiment is included in the average shown in column #7 of (B). D) Fluorescence micrographs show co-clustering of C-terminally labeled STIM1(1–462, L251S) and STIM1(del302–448) after addition of ATP/TG. STIM1 constructs were expressed at moderate levels in COS7 cells without Orai1 co-expression. Scale bar is 10 µm.

For further characterization of the apparent conformational extension in STIM1 caused by store depletion, we used the FRET acceptor STIM1 (del302–448)-mApple as a reference, and we compared sequential truncations of donor-labeled STIM1 (Figure 4A). As summarized in Figure 4B for multiple experiments, we first confirmed that deleting the identical sequence in the donor construct, STIM1(del302–448)-AcGFP, as in the acceptor constructs results in no stimulated change in FRET between these symmetrically placed donor and acceptor probes (Figure 4B, column 2). This is consistent with a previous study [26] and indicates that the absence of CAD/SOAR + CC1α3in both constructs prevents stimulated oligomerization. Truncation of the donor construct just prior to CAD/SOAR (STIM1(1–343)-AcGFP) results in almost no stimulated FRET change (Figure 4B, column 4), consistent with the view that the stimulated decrease in FRET observed with wt STIM1-AcGFP as donor (Figure 4B, column 3; Figure 3C, upper right) is due to a conformational extension in the cytoplasmic segment of wt STIM1. We also observe no significant FRET change upon stimulation when the donor construct is truncated just after CAD/SOAR (STIM1(1–448)-AcGFP; Figure 4B, column 5), suggesting that this construct also does not undergo a stimulated conformational extension. This observation is consistent with STIM1(1–448)-AcGFP already having an extended structure corresponding to the activated conformation, exhibiting spontaneous cluster formation (Figure 1B, lower middle pane l) and constitutively elevated basal Ca2+ levels (Figure 1C). Importantly, we found that extending the donor sequence to 462 (STIM1(1–462)-AcGFP), such that the additional 14 residues just C-terminal to CAD/SOAR are included, restores nearly all of the stimulated decrease in FRET that is observed with the wt donor (Figure 4B, compare columns 3 and 6). This result provides additional evidence that this 14-residue sequence (449–462) contributes significantly to the interactions that maintain the cytoplasmic CAD/SOAR-containing segment of STIM1 in an inactive conformation in the absence of stimulated store depletion.

Previous studies showed that the CRAC-activating mutation L251S causes a conformational change in the cytoplasmic segment of STIM1 to a more extended structure [9, 10]. We evaluated whether this activating mutation in STIM1(1–462)-AcGFP interferes with the observed decrease in FRET to STIM1 (del302–448)-mApple upon stimulation (Figure 4B, column 6). Confocal images show that STIM1 (1–462, L251S)-AcGFP and STIM1 (del302–448)-mApple co-localize in large domains at the ventral surface of these cells (Figure 4D), and this appearance is similar with or without stimulation by ATP+TG. As shown in a representative FRET experiment in Figure 4C and summarized for multiple experiments in Figure 4B, column 7, the L251S mutation prevents the stimulated decrease in FRET observed for STIM1 (1–462)-AcGFP as donor. These results are consistent with the view that this activated mutant STIM1 (1–462, L251S)-AcGFP has an active, extended structure that promotes oligomerization, and does not undergo a further conformation change in response to store depletion.

## 4. Conclusion

Our study with live cells provides evidence for a conformational extension in the cytoplasmic segment of STIM1 that is stimulated by depletion of Ca^2+^ from ER stores. We further identify a 14 amino acid sequence (449–462) adjacent to the C-terminal end of CAD/SOAR that is important for maintaining STIM1 in an inactive conformation in the absence of store depletion. Our initial experiments showed that truncation of the cytoplasmic segment of STIM1 just after CAD/SOAR (STIM1(1–448)) results in spontaneous, microscopically visible clustering of this construct and elevated basal cytosolic Ca^2+^ levels in resting COS7 cells. C-terminal addition of the 14 amino acids just after CAD/SOAR (STIM1(1–462)) is sufficient to prevent spontaneous clustering in resting cells and elevated basal cytosolic Ca^2+^, such that basal Ca^2+^ with STIM1(1–462) is similar to that with wt STIM1 (Figure 1). Truncation of STIM1 just after CAD/SOAR (STIM1(1–448)) reduces, but does not prevent, an increase in FRET between these constructs that were symmetrically labeled with donor and acceptor probes at N- or C-termini (Figure 2). This FRET increase corresponds to oligomerization of STIM1, and the reduction in stimulated FRET observed with STIM1(1–448) compared to wt STIM1 or STIM1(1–462) is likely due to partial oligomerization of STIM1(1–448) prior to stimulated store depletion.

Our subsequent experiments evaluated FRET between C-terminal donor and acceptor probes located in different full length, deletion, and truncation constructs. We monitored stimulated FRET from wt STIM1-AcGFP to STIM1(del302–448)-mApple in which the CC1α3 and CAD/SOAR sequences are deleted, and we observed a decrease in FRET caused by store depletion, despite microscopically visible co-clustering of these asymmetric constructs under these conditions (Figure 3). We found that this stimulated decrease in FRET from the wt donor construct depends on deletion of CAD/SOAR but not CC1α3 from the acceptor construct. The stimulated decrease in FRET is substantially reduced by additional deletion of the 14 amino acid sequence (449–462) in the acceptor construct (STIM1 (del302–462)-mApple) (Figure 3C & D). To account for this stimulated decrease in FRET from wt STIM1-AcGFP to STIM1(del302–448)-mApple, we propose that store depletion activates a transition to a more extended conformation in wt STIM1 that does not occur to the same extent in the CAD/SOAR-deleted construct. To test this hypothesis, we used STIM1 (del302–448)-mApple as the reference acceptor construct, and we compared FRET measurements for a series of sequentially truncated STIM1 donor constructs. We found that the stimulated decrease in FRET depends on a donor STIM1 sequence that includes the 14 amino acid sequence (449–462) immediately following CAD/SOAR, consistent with a role for this sequence in the more compact conformation of STIM1 in the unactivated state (Figure 4). We also found that introduction of the constitutively activating mutation, L251S, into the donor construct that is sufficient for the stimulated decrease in FRET, STIM1(1–462)-AcGFP, prevents the decrease in FRET caused by store depletion; for this case, CC1 is already conformationally extended [9,10].

**Figure 5:**
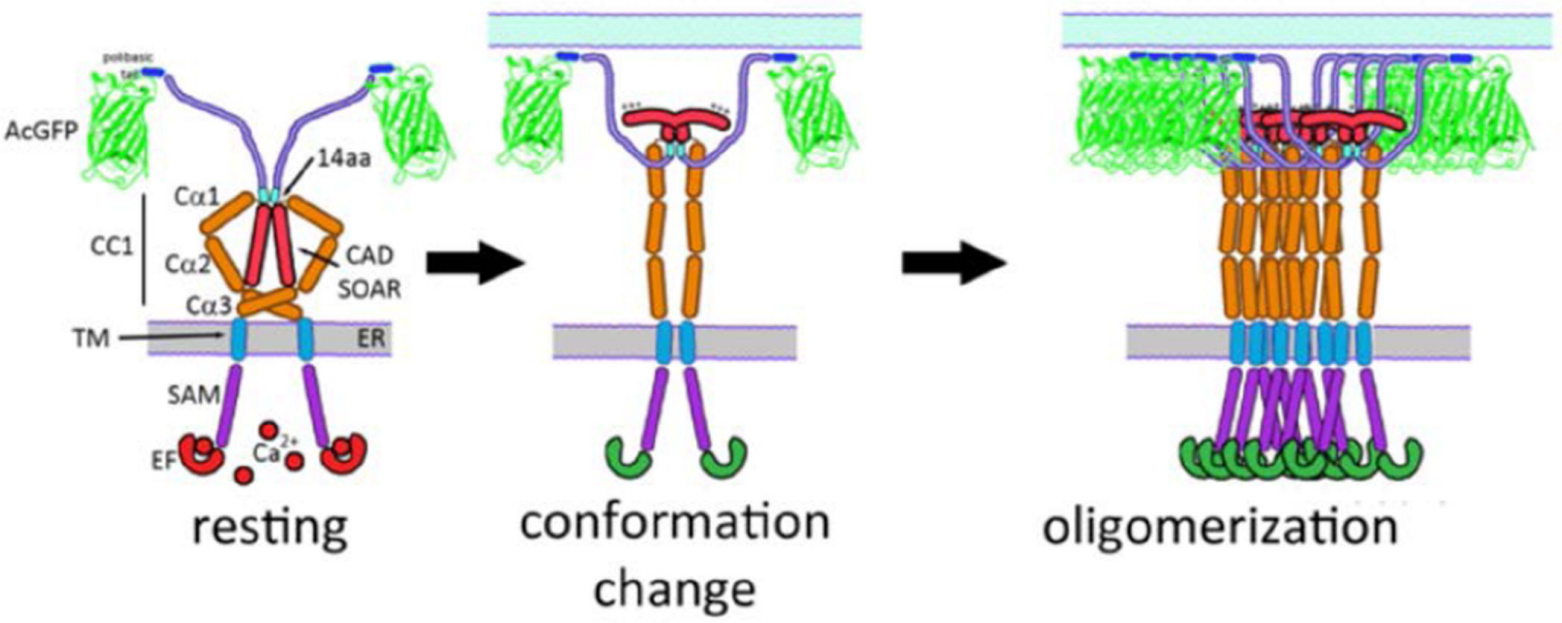
Model for structures of STIM1 before (left) and after (middle and right) stimulation by Ca^2+^ depletion of ER stores, based on this study. Segments in unstimulated STIM1 (left) are labeled as in Figure 1A. As characterized in this study, the conformation of STIM1 is regulated (in part) by a 14 amino acid sequence (449–462). Stimulation causes the interactions of this sequence to be altered, resulting in a conformational extension accompanied by oligomerization of STIM. Consequent exposure of positively charged sequences in CAD/SOAR facilitates coupling with Orai1 to initiate SOCE.

Our results are consistent with a model in which the basic sequence in CAD/SOAR (382–386) that is critical for activation of SOCE [8,16,17] is sequestered from association with Orai1 at the plasma membrane in unstimulated cells (Figure 5, left panel). This basic sequence becomes accessible for association with the C-terminal acidic sequence in Orai1 [11,27,29] due to a conformational transition in STIM1 that is stimulated by Ca^2+^ depletion from ER stores (Figure 5, middle panel). Oligomerization of constitutively dimeric STIM1 [6,8,11,22,30,31] that is necessary for stoichiometric coupling to hexameric Orai1 [7] accompanies this conformational transition in the cytoplasmic segment of STIM1 (Figure 5, right panel).

Using synthetic transmembrane STIM1 constructs, Fahrner et al. recently provided evidence that CC1α1 (238–271) association with CC3 (400–450) contributes to the inactive state of STIM1 in the absence of store depletion [32]. They proposed a model for the resting conformation of STIM1 that is similar to our model depicted in Figure 5, left panel. In our model, the 14 amino acid sequence (449–462) that we have identified as contributing to this resting conformation is proximal to the CC1α3 segment of STIM1 (308–337), suggesting that these two segments may associate to mediate regulation of STIM1 activation. The extended conformation of stimulated full length STIM1 that we infer from FRET measurements between wt STIM1-AcGFP and STIM1(del302–448)-mApple or STIM1(del344–448)-mApple (Figure 3) is consistent with evidence from in vitro measurements of soluble STIM1 cytoplasmic fragments in which activating mutations cause a more extended conformation in this region as detected by FRET and luminescence RET [9, 10]. Our findings provide evidence in live cells that store depletion stimulates a conformational extension in the cytoplasmic segment of STIM1 corresponding to the activated state.

In previous models, the C-terminal polybasic sequence is commonly depicted in contact with the ER membrane in unstimulated cells [10,32,33]. However, it is clear from images of cells with over-expression of wt STIM1-AcGFP (Figure 1B) and related constructs in the absence of Orai1 that the C-terminal polybasic sequence preferentially associates with the plasma membrane, even in the absence of stimulation [22,24,25]. The presence of this polybasic sequence in wt STIM1-AcGFP may account for its enhanced extension upon stimulation compared to STIM1(1–462)-AcGFP, as reflected in reduced FRET from the latter to STIM1(del302–448)-mApple (Figure 4B).

Our results further indicate a critical role for the 14 amino acid sequence (449–462) just C-terminal to CAD/SOAR in mediating the inactive conformation of STIM1 prior to stimulation, and thereby preventing spontaneous coupling to Orai1. The absence of this sequence in the CAD/SOAR-deleted acceptor construct (STIM(del302–462)-mApple) substantially reduces the magnitude of the stimulated decrease in FRET from wt STIM1-AcGFP (Figure 3C and D). This result is consistent with this acceptor construct existing in an extended conformation prior to stimulation, and it is consistent with the 14 amino acid sequence contributing to maintaining STIM1 in a more compact, inactive conformation. Confirming this view, the 14 amino acid sequence is a necessary part of the donor construct that gives rise to the stimulated decrease in FRET to the reference CAD/SOAR-deleted acceptor (STIM1(del302–448)-mApple) (Figure 4B, columns 5 and 6). These results indicate that the stimulated decrease in FRET observed with the donor construct (STIM1(1–462)-AcGFP) depends on a conformation change that is limited or does not occur in (STIM1(1–448)-AcGFP) during store depletion. With qualitatively similar results for the wt STIM1 donor construct (Figure 4B, column 3), the 14 amino acid sequence in STIM1 is shown to be critical for the store depletion-dependent release of CAD/SOAR that leads to the structural extension and stimulated decrease in FRET observed. These stimulated conformational changes facilitate exposure of CAD/SOAR for functional coupling with Orai1 at the plasma membrane.

It is not yet clear what this 14 amino acid sequence associates with to stabilize the inactive conformational state of STIM1. Association with CC1α3 is suggested by the structural proximity reported for *C. elegans* CC1α3/SOAR [15]. A longer C-terminal sequence following CAD/SOAR (445–475) was previously reported to regulate SOCE [12], and binding to the protein SARAF(26) by a more extended sequence (448–530) has also been suggested as a possible mechanism for regulation, as has Ca^2+^ binding to an inactivation domain (475–483) [34–36]. However, as described above, the sequence (449–462) in STIM1(1–462) is sufficient to prevent spontaneous clustering and basal elevation of cytoplasmic Ca^2+^ that is observed with STIM1(1–448) (Figure 1B and C). Elimination of this 14 amino acid sequence in the CC1α3/CAD-deleted acceptor (STIM1(del302–462)mApple) causes a reduction in the stimulated FRET change that reflects the conformational extension of the wt donor (STIM1-AcGFP) (Figures 3D), and its elimination in the donor construct (STIM1(1–448)AcGFP) abrogates the FRET-detected conformational extension (Figure 4B). Although this 14 amino acid sequence (449–462) in STIM1 could associate in *trans* with CC1α3 (308–337) in the unstimulated STIM1 dimer, this is not likely to participate in the regulation of a conformational extension. This same (449–462) sequence in the donor is clearly important for maintaining it in an inactive state prior to stimulated store depletion, even when the acceptor construct lacks CC1α3 (Figure 4B). Furthermore, use of STIM1(del302–448)-mApple as a reference acceptor construct consistently revealed a conformation change occurring in donor constructs containing the (449–462) sequence. Thus, the 14 amino acid sequence STIM1 (449–462) appears to play a critical role in maintaining the inactive state prior to stimulation, even when the interacting STIM1 partner in the dimer pair does not contain CC1α3. In future experiments, it may be instructive to further evaluate the role of this 14 amino acid sequence by its deletion from the intact STIM1 protein, or by selective mutagenesis of specific residues in this sequence to alanines.

In summary, our results with live cells substantially extend previous evidence, both with isolated fragments in solution [9,10] and with synthetic transmembrane constructs transfected into cells [32]: an activating conformational change in the cytoplasmic segment of STIM1 results from depletion of intracellular Ca^2+^ stores. Our measurements of increased FRET between both C- and N-terminally labeled symmetric STIM1 constructs are consistent with oligomerization that accompanies this stimulation, and a conformational extension is revealed by the stimulated decrease in FRET measured between a STIM1 that is donor-labeled at its C-terminus and CAD/SOAR-deleted STIM1 that is acceptor-labeled at its C-terminus. Furthermore, our results identify a 14 amino acid sequence just C-terminal to CAD/SOAR that is necessary for maintaining STIM1 in an inactive conformation until store depletion activates both conformation extension and oligomerization leading to exposure of CAD/SOAR for functional coupling to Orai1.

## Supplementary Material

supplemental

## Figures and Tables

**Table 1:** Constructs and primer sequences used in this study.

Construct name	Primer sequence (restriction sites underlined)	
TK-Orai1 (PCR amplification)	fwd	3’-TATAGCTAGCTCCATGCATCCGGAGCCCG-5’
	rev	3’-TATAGCGGCCGCTAGGCATAGTGGCTGCCGGGC-5’
STIM1(1–462)AcGFP and STIM1(1–462)mApple (mutagenesis deletion)	fwd	3’-TAGACCCCAGCTGGATGGGCCGAATTCTGCAGTCGA CGGTAC-5’
	rev	3’- GTACCGTCGACTGCAGAATTCGGCCCATCCAGCTGG GGTCTA-5’
STIM1(1–448)AcGFP and STIM1(1–448)mApple (mutagenesis deletion)	fwd	3’- AACAACCCTGGCATCCACGGAATTCTGCAGTCGACG GTACC-5’
	rev	3’ ACTGCAGAATTCCGTGGATGCCAGGGTTGTTGACAAT CTGGAAGCC-5’
STIM1(1–343)AcGFP and STIM1(1–343)mApple (mutagenesis deletion)	fwd	3’- AATCTCACAGCTCATGGTATGCTCGAATTCTGCAGTC GACGGTACC-5’
	rev	3’- TACCGTCGACTGCAGAATTCGAGCATACCATGAGCT GTGAGATTCTAGC-5’
STIM1(del.302–448)AcGFP and STIM1(del.302– 448)mApple (mutagenesis deletion)	fwd	3’- AAGCCCAGCGGCTGAAGTCACTGGTGGCTGCC-5’
	rev	3’- GGCAGCCACCAGTGACTTCAGCCGCTGGGCTT-5’
STIM1(del.302– 462)mApple (mutagenesis deletion)	fwd	3’-TAAGCAGGAAGCCCAGCGGCTGAAGAGTACACGCCC CAACCCTGCT-5’
	rev	3’- AGCAGGGTTGGGGCGTGTACTCTTCAGCCGCTGGGC TTCCTGCTTA-5’
STIM1(del.344– 448)mApple (mutagenesis deletion)	fwd	3’-AATCTCACAGCTCATGGTCACTGGTGGCTGCCC TCAACATAGACC-5’
	rev	3’- TATGTTGAGGGCAGCCACCAGTGACCATGAGCT GTGAGATTCTAGCTCCTTCTCTG-5’

## Abbreviations

ER: endoplasmic reticulum
SOCE: store-operated Ca^2+^ entry
CAD/SOAR: CRAC activation domain
Ca^2+^: calcium
PBS: phosphate buffered saline
FRET: fluorescence resonance energy transfer
CRAC: calcium release-activated calcium
PM: plasma membrane
BBS: buffered saline solution
TG: thapsigargin
