# Supplementary material for: STIM1 activation is regulated by a 14 amino acid sequence adjacent to the CRAC activation domain
Source: AIMS Biophys. Author manuscript; Available in PMC 2016 May 27. (PMC4883682; doi:10.3934/biophy.2016.1.99)
Supplement: supplemental [file NIHMS765388-supplement-supplemental.docx]

***Research article***

**STIM1 activation is regulated by a 14 amino acid sequence adjacent to the CRAC activation domain**

**Marek K. Korzeniowski, Barbara Baird, and David Holowka***

From the Department of Chemistry and Chemical Biology Cornell University. Ithaca, NY 14853

*** Correspondence:** Email: e-mail: dah24@cornell.edu; Tel: 607-255-6140.

**Supplementary materials**


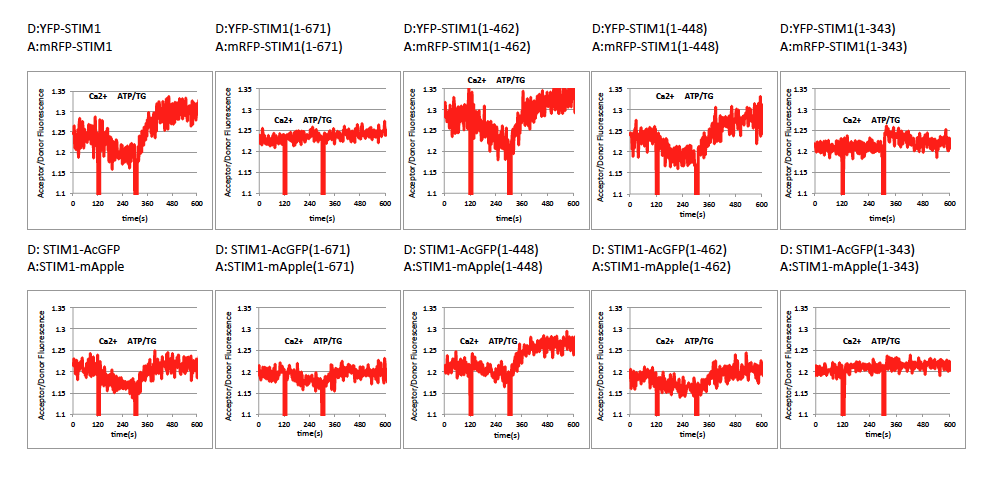


**Figure S1.** Representative raw data showing the ratio of acceptor to donor fluorescence as a measure of sensitized FRET emission for the normalized FRET responses shown in Figure 2B, as well as for stimulated FRET responses with C-terminally truncated mutants. Experiments were carried out similarly to those in Figure 2.

**Figure S2.** Chemical crosslinking of COS7 cells expressing constructs indicated shows evidence for the existence of STIM1 dimers in the absence of stimulation for all three deletion mutants used in this study. Left hand panel shows unreduced crosslinked STIM1 constructs and right hand panel shows the same samples following reduction with **β**-ME to cleave the chemical crosslinks, all detected in whole cell lysates by an anti-STIM1 Ab. Chemical crosslinking was carried out using dithiobis(succinimidyl propionate) (DSP) at 100 **μ**M for 30 min at room temperature, followed by quenching and lysis in sample buffer. Right-most lane shows molecular weight standards.
